# Regulatory T cells contribute to the immunosuppressive phenotype of neutrophils in a mouse model of chronic lymphocytic leukemia

**DOI:** 10.1186/s40164-023-00452-9

**Published:** 2023-10-10

**Authors:** Agnieszka Goral, Marta Sledz, Aneta Manda-Handzlik, Adrianna Cieloch, Alicja Wojciechowska, Mieszko Lachota, Agnieszka Mroczek, Urszula Demkow, Radoslaw Zagozdzon, Katarzyna Matusik, Malgorzata Wachowska, Angelika Muchowicz

**Affiliations:** 1 Department of Immunology, Medical University of Warsaw, Warsaw, 02-097 Poland; 2 Department of Clinical Immunology, Medical University of Warsaw, Warsaw, 02-097 Poland; 3 Present Address: Doctoral School, Medical University of Warsaw, Warsaw, 02-091 Poland; 4 Department of Laboratory Diagnostics and Clinical Immunology of Developmental Age, Medical University of Warsaw, Warsaw, 02-091 Poland; 5 Department of Ophthalmology, Children’s Memorial Health Institute, Warsaw, 04-730 Poland

**Keywords:** CD62L, Chronic lymphocytic leukemia (CLL), Neutrophils, Regulatory T cells (Treg), Immunosuppression

## Abstract

**Background:**

Impaired neutrophil activity is an important issue in chronic lymphocytic leukemia (CLL), as it contributes to a dysfunctional immune response leading to life-threatening infections in patients. Some features typical of CLL neutrophils, e.g., the B-cell-supportive secretion profile, have already been described. However, most of these studies were performed on cells isolated from peripheral blood. It is still unclear which molecular factors and cell types are involved in shaping neutrophil function and phenotype in the CLL microenvironment. Since regulatory T cells (Treg) play an important role in CLL progression and influence the activity of neutrophils, we investigated the crosstalk between Treg and neutrophils in the spleen using a murine model of CLL.

**Methods:**

In this work, we used an Eµ-TCL1 mouse model of human CLL. For our in vivo and ex vivo experiments, we inoculated wild-type mice with TCL1 leukemic cells isolated from Eµ-TCL1 transgenic mice and then monitored disease progression by detecting leukemic cells in peripheral blood. We analyzed both the phenotype and activity of neutrophils isolated from the spleens of TCL1 leukemia-bearing mice. To investigate the interrelation between Treg and neutrophils in the leukemia microenvironment, we performed experiments using TCL1-injected DEREG mice with Treg depletion or RAG2KO mice with adoptively transferred TCL1 cells alone or together with Treg.

**Results:**

The obtained results underline the plasticity of the neutrophil phenotype, observed under the influence of leukemic cells alone and depending on the presence of Treg. In particular, Treg affect the expression of CD62L and IL-4 receptor in neutrophils, both of which are crucial for the function of these cells. Additionally, we show that Treg depletion and IL-10 neutralization induce changes in the leukemia microenvironment, partially restoring the “healthy” phenotype of neutrophils.

**Conclusions:**

Altogether, the results indicate that the crosstalk between Treg and neutrophils in CLL may play an important role in CLL progression by interfering with the immune response.

**Supplementary Information:**

The online version contains supplementary material available at 10.1186/s40164-023-00452-9.

## Background

Neutrophils are one of the key myeloid-derived populations present in the tumor microenvironment and are frequently involved in tumor growth, angiogenesis, metastatic formation and immunosuppression [1, 2]. The diversity of the roles that neutrophils play in the neoplastic process results from their remarkable ability to respond to many different factors and cytokines that may also alter their functions [3]. In response to transforming growth factor (TGF)-β, interferon (IFN)-β, or interleukin (IL)-35, neutrophils shift from the N1 to the N2 phenotype and support tumor progression [4]. In particular, the protumor actions of neutrophils occur due to the production of proangiogenic factors (vascular endothelial growth factor, VEGF; prokineticin 2, PROK2; matrix metallopeptidase 9, MMP9), inhibition of the immune response (indoleamine 2,3-dioxygenase, IDO; arginase, ARG1), and stimulation of tumor cell proliferation (MMP9, elastase) [1, 4, 5]. Remarkably, depending on the type of neoplastic disease, the phenotype and functions of neutrophils appear different. The commonly used division into N1 and N2 neutrophils does not fully define the changes that occur in these cells during the neoplastic process. Apparently, the functions of neutrophils described in hematological malignancies are complex and differ from those observed in solid tumors. Indeed, neutrophils are able to support leukemia progression through mechanisms that mimic the cooperation of neutrophils with healthy regulatory B cells [6].

The progression of chronic lymphocytic leukemia (CLL) is strongly associated with the modification of diverse immune cell populations to create a niche appropriate for the proliferation and survival of leukemic B cells. Importantly, CLL cells are completely dependent on the microenvironment [7]. Thus, when cultured alone without special treatment, CLL cells do not proliferate under in vitro conditions. The immunosuppressive CLL microenvironment supports disease progression, contributes to CLL escape from immune surveillance and significantly destabilizes the whole immune response of a CLL patient [8]. The majority of studies concerning CLL focus on dysfunctions of the adaptive immune response, particularly T-cell exhaustion and regulatory T-cell-derived immunosuppression [9]. Additionally, defects in the complement system and hypogammaglobulinemia were identified as major mechanisms of immune system dysregulation that reduce the outcome of applied therapies [10, 11]. Consequently, CLL patients do not respond effectively to vaccines and suffer from infections that significantly increase CLL mortality rates [12]. In light of this study, it is worth emphasizing that neutropenia and the limited response of neutrophils against bacteria are also common in CLL patients [13]. In a mouse model of CLL, neutrophil functions are substantially altered, as they are more resistant to apoptosis and produce A proliferation-inducing ligand (APRIL) and B-cell-activating factor (BAFF), and are more prone to release neutrophil extracellular traps (NETs) that support the survival and proliferation of leukemic cells [14]. All these data support the importance of neutrophils in leukemia development and progression, underlining neutrophil plasticity in response to various mediators released in the leukemia microenvironment [15]. Moreover, recent studies show that neutrophils can cooperate with both innate and adaptive components of the immune response. The crosstalk of neutrophils with other cell populations leads to effective eradication of pathogens and tissue repair or may polarize neutrophils into an immunosuppressive subpopulation [16–18]. The changes in neutrophil phenotype were shown to be associated with IL-10/TGF-β production by leukemic cells. However, IL-10 and TGF-β are also important players in the secretion profile and functions of regulatory T cells (Treg).

Eµ-TCL1 mice are a model of CLL used worldwide. TCL1 leukemia-bearing mice (both transgenic and wild-type mice inoculated with TCL1 leukemic cells) develop the disease, which to a great extent reflects human CLL in terms of clinical characteristics, molecular features of malignant B cells and the immunosuppressive microenvironment [19–21]. In our previous study, we defined a specific, leukemia-associated Treg population in a TCL1 mouse model. We observed that depletion of Treg triggers complete leukemia eradication [22]. The specific CD25^lo^CD44^lo^ and LAG3^+^ CLL Treg subpopulations express IL-10, which together with IL-4 may escalate the immunosuppressive activity of neutrophils in the tumor microenvironment [23–25]. Therefore, in this study, we analyzed the phenotype and functions of neutrophils isolated directly from the leukemia microenvironment of TCL1 leukemia-bearing mice. In particular, we demonstrate that by overcoming the immunosuppressive environment and Treg depletion, it is possible to reverse the changes in the phenotype of CLL-associated neutrophils. Remarkably, our data indicate that the presence of Treg in the CLL microenvironment is important for neutrophil loss of function and changes in phenotype.

## Materials and methods

### Reagents

Diphtheria toxin (DT) from *Corynebacterium diphtheriae* (Sigma-Aldrich, St Louis, MA, USA) was aliquoted and stored at -80 °C. The CM-H_2_-DCFDA fluorescent probe (Molecular Probes, Eugene, OR, USA) was resuspended in dimethyl sulfoxide (DMSO) (Sigma-Aldrich) to a 1 mM concentration, aliquoted and stored at -20 °C. InVivoMAb anti-mouse IL-10 antibody (JES5-2A5 clone) and InVivoMAb rat IgG1 isotype control, anti-horseradish peroxidase (both from BioXcell, Lebanon, NH, USA) were stored at 4 °C. Mouse GM-CSF (415-ML) was from R&D Systems (Minneapolis, MN, USA), mouse C5a (HC1101) were purchased from Hycult Biotech (Uden, The Netherlands), and cytochalasin B (C6762) from *Drechslera dematioidea* was purchased from Sigma-Aldrich.

### Animals

In vivo studies were planned and carried out according to both the EU Directive 2010/63/EU and the Polish legislation for animal experiments of the Polish Ministry of Science and Higher Education (February 26, 2015). The experiments were performed in the Animal Facility of the Medical University of Warsaw upon approval by the Second Local Ethics Committee for Animal Experimentation in Warsaw. Six- to 12-week-old female or male (never mixed in one experiment) mice of the following strains were used for the study: C57BL6/J (WT) (Mossakowski Medical Research Centre), C57BL/6-Tg(Foxp3-DTR/EGFP)23.2Spar/Mmjax (DEREG) (Medical University of Warsaw) and B6(Cg)-Rag2tm1.1Cgn/J (RAG2KO) (Medical University of Warsaw). For the model of CLL, the mice were injected intravenously (i.v.) with 5 × 10^6^ splenocytes or leukemic CD5^+^ CD19^+^ TCL1 cells isolated from the spleen of Eµ-TCL1 transgenic mouse. The cells used for the adoptive transfer were previously propagated in C57BL6/J WT mice once or maximally twice.

### In vivo experiments

Leukemia progression in mice upon adoptive transfer was assessed based on the percentage of leukemic TCL1 cells (CD5^+^ CD19^+^) among white blood cells in the peripheral blood samples collected from the cheek vein into EDTA-coated tubes (Microvette 500 K3E, Sarstedt AG & Co., Nümbrecht, Germany) and analyzed by flow cytometry. For depletion of regulatory T cells, DEREG mice were treated with DT (50 µg/kg) administered intraperitoneally (i.p.) every four days. Anti-IL-10 antibody or the appropriate isotype control was administered i.p. every third day at a dose of 100 µg/mouse. RAG2KO mice were inoculated i.v. with 5 × 10^6^ TCL1 CD19^+^ cells alone or together with 2 × 10^5^ GFP^+^ Treg sorted from the spleens of TCL1 leukemia-bearing DEREG mice. After indicated point of time, the animals were sacrificed, and spleens were isolated for further analysis. The schemes of the in vivo experiments are presented in detail in the appropriate figures.

### Cell isolation

For a single-cell suspension of splenocytes, the isolated spleens (SPL) were cut into small pieces with scissors and then passed through a 150 μm cell strainer using syringe plungers. CD19^+^ and neutrophil cell subpopulations were isolated by immunomagnetic selection using the EasySep™ Mouse B Cell Isolation Kit and EasySep™ Mouse Neutrophil Enrichment Kit (STEMCELL Technologies, Vancouver, Canada), respectively, according to the manufacturer’s protocols. The purity of the isolated populations was over 90% for CD19^+^ cells and approximately 70–80% for neutrophils. For isolation of bone marrow Ly6G^+^ cells, hindlimbs of healthy, 5- to 6-week-old C57BL6/J WT donors were detached, and femurs were separated. The bone marrow from the femurs was rinsed out with phosphate buffered saline (PBS) with 2% fetal bovine serum (FBS) and 1 mM EDTA using a syringe. Next, neutrophils were isolated from the cell suspension using an EasySep™ Mouse Neutrophil Enrichment Kit (STEMCELL Technologies).

### Flow cytometry

A single-cell suspension of splenocytes was prepared as described above, and then, to remove red blood cells, the splenocytes were suspended in ACK Lysing Buffer (Thermo Fisher Scientific, Waltham, MA, USA) for 3–4 min at room temperature (RT) and washed with PBS. Next, the white blood cells were stained with a Zombie NIR™ Fixable Viability kit or Zombie Violet™ Fixable Viability Kit (BioLegend, San Diego, CA, USA) for 20 min at RT and washed with PBS. For blocking of nonspecific binding sites, the cells were incubated with purified rat anti-mouse CD16/CD32 (Mouse BD Fc Block™; clone 2.4G2, BD Biosciences, La Jolla, CA, USA) for 15 min at RT and subsequently stained for surface markers with proper fluorochrome-conjugated antibodies (all antibodies used in this study are listed in Supp. Table 1) for 20–30 min at RT. For intracellular staining, the cells were treated with BD Cytofix/Cytoperm™ Fixation and Permeabilization Solution (BD Biosciences) and then stained with appropriate fluorochrome-conjugated antibodies to detect the intracellular markers of interest. After final washing with PBS, the cells were analyzed using a BD FACSCanto™ II Flow Cytometer and BD FACS Diva Software (v8.0.1) (BD Biosciences). For further analyses, FlowJo Software (v. 10.6.1) (FlowJo LLC, Ashland, OR, USA) was used.

### Sorting of regulatory T cells

CD4^+^ GFP^+^ Treg were sorted from the spleens of TCL1 leukemia-bearing DEREG mice. Prior to sorting, the CD4^+^ cell subpopulation was enriched. For that, the isolated splenocytes were subjected to immunomagnetic positive selection for CD19^+^ cells using the EasySep™ Mouse CD19 Positive Selection Kit II (STEMCELL Technologies), and then, the negative fraction, devoid of leukemic CD19^+^ cells, was subjected to negative selection using the EasySep™ Mouse CD4^+^ T cell Isolation Kit (STEMCELL Technologies) according to the manufacturers’ instructions. The CD4^+^ GFP^+^ Treg were sorted using a BD FACSAria™ III Cell Sorter (BD Biosciences).

### Detection of intracellular ROS levels using CM-H_2_-DCFDA

Splenocytes isolated from control and TCL1 leukemia-bearing mice were suspended at a density of 4 × 10^6^ cells/ml, incubated with 1.0 µM CM-H_2_-DCFDA fluorescent probe (Molecular Probes, Eugene, OR, USA) in PBS at 37 °C for 30 min and then washed with PBS. Next, for distinguishing the population of living granulocytes, the cells were incubated with a Zombie Aqua™ Fixable Viability Kit (BioLegend) for 20 min, washed with PBS and subsequently stained with the following fluorochrome-conjugated antibodies: anti-CD11b-PE (eBiosciences, San Diego, CA, USA), anti-Ly6C-PerCP-Cy5.5 and anti-Ly6G-APC-Cy7 (both from BD Bioscience). After a final wash with PBS, to determine the intracellular ROS levels, the geometric mean fluorescence intensity (MFI) of oxidized CM-H_2_-DCFDA in CD11b^+^ Ly6G^+^ cells was analyzed by flow cytometry.

### Degranulation of neutrophils

A total of 5 × 10^5^ neutrophils were primed with 100 ng/mL murine GM-CSF for 15 min, and 5 µg/mL cytochalasin B was added for 5 min. Then, the cells were activated by 100 nM murine C5a for 15 min. After this, the reaction was stopped with ice-cold PBS. The supernatant was discarded, and the cells were labeled with anti-CD63 PE antibody. CD63 expression on the cell surface was analyzed with a BD LSR Fortessa flow cytometer.

### Phagocytosis assay

Neutrophils (3 × 10^5^) were incubated with 25 µg of *Escherichia coli* bioparticles for 30 min at 37 °C and 5% CO_2_. Subsequently, trypan blue at a final concentration of 0.01% was added to quench the fluorescence of nonphagocytosed bioparticles. Then, the cells were washed, and the percentage of cells phagocytosing *E. coli* bioparticles was analyzed with a BD LSR Fortessa flow cytometer.

### Neutrophil – splenocyte coculture

Freshly isolated neutrophils (1 × 10^6^, bone marrow Ly6G^+^) were seeded into 24-well plates alone, with splenocytes from healthy donors (SPL) or from TCL1 leukemia-bearing mice (TCL1) at a 1:2 ratio. For exclusion of cell‒cell interactions, neutrophils were separated from splenocytes using a Transwell. α-IL-10 or isotype control (ISO) antibody (Bio X Cell, Lebanon, NH, USA), at a final concentration of 10 µg/ml, was added. Upon 19 h of incubation, Transwells with splenocytes were removed from culture plates. The neutrophils were rinsed with cold PBS (Corning, Glendale, Arizona, USA) and transferred to tubes. Approximately ¼ of each sample was stained with 1 µg/ml propidium iodide (Thermo Fisher Scientific) in PBS and analyzed immediately by flow cytometry. The remains of the samples were blocked for nonspecific binding sites and stained with antibodies for extracellular markers CD11b, IL-4R, Ly6G, Ly6C and CD62L and analyzed by flow cytometry as described above.

### RNA-seq data analysis

The entire analysis was performed in R (version 4.1.2) using RStudio [26, 27]. RNA isolation, sequencing and data processing were performed as previously described in Goral et al. [22]. First, the previously generated pseudocount data were loaded and filtered using the “data.table” and “dplyr” R packages [28, 29]. The analysis was limited to genes encoding receptor ligands. Then, the data were scaled, clustered using “dendsort” and visualized with “ComplexHeatmap” [30, 31]. The table with previously computed differentially expressed genes was created with “flextable” [32].

### Statistical analysis

GraphPad Prism 6 Software (GraphPad Software, Inc., San Diego, CA, USA) was used for data analysis. Statistical significance was evaluated by the Mann‒Whitney U test.

## Results

### Neutrophils isolated from the spleens of TCL1 leukemia-bearing mice have functional impairment and an altered phenotype

**Fig. 1:**
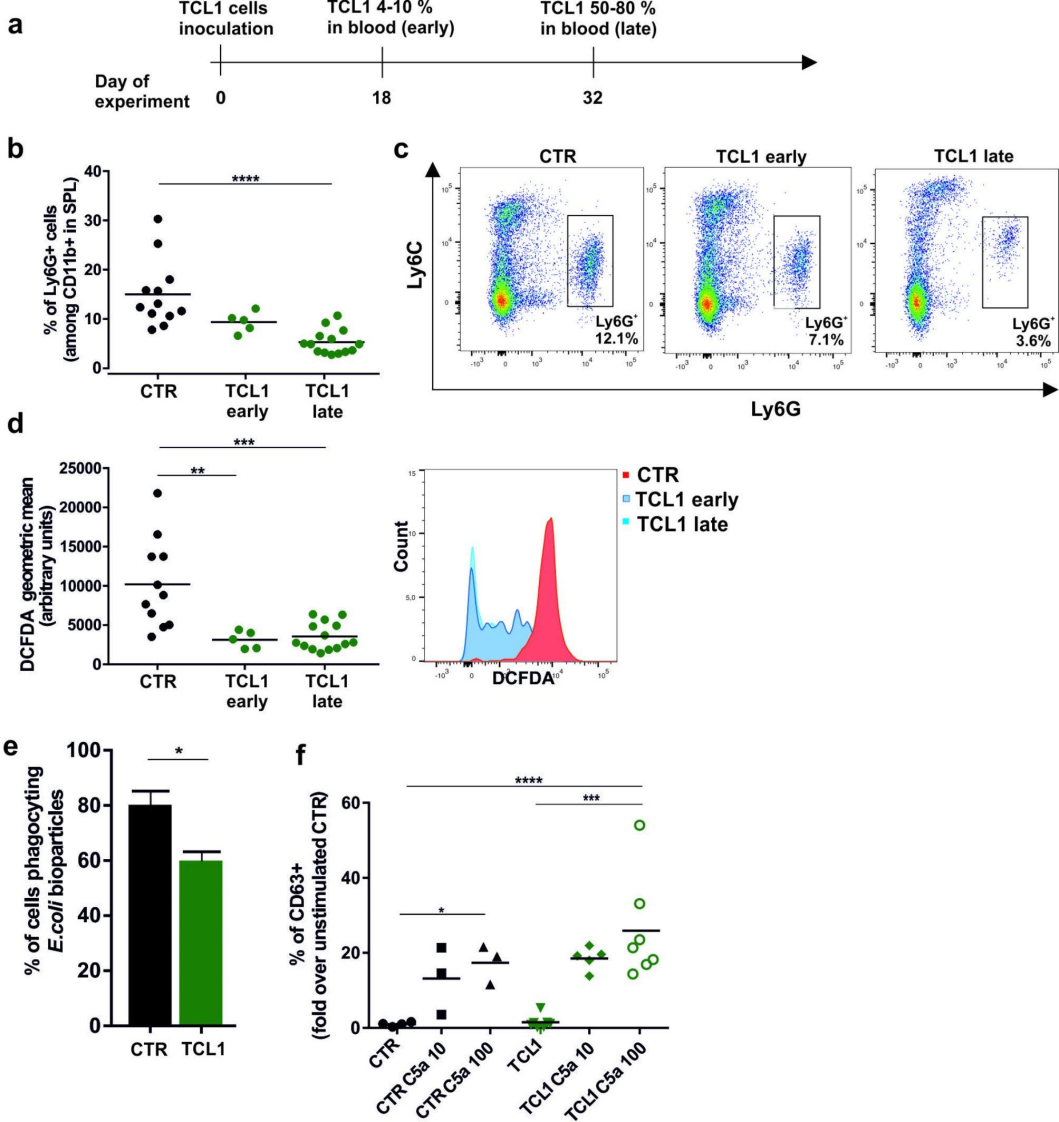
Dysfunction of neutrophils in TCL1 leukemia-bearing mice. **a** Scheme presenting the timeline of the experiment. Wild-type C57BL6/J mice were injected with splenocytes isolated from Eµ-TCL1 transgenic mouse. Next, leukemia progression was assessed by flow cytometry based on the percentage of leukemic TCL1 cells (CD5^+^ CD19^+^) among white blood cells (WBC) in the peripheral blood. The function of splenic granulocytes was analyzed at two stages of disease development: 4–10% (TCL1 early) and/or 50–80% (TCL1 late) of CD5^+^ CD19^+^ cells among WBCs in the blood. **b** The percentage of granulocytes (Ly6G^+^) among all myeloid (CD11b^+^) cells assessed by flow cytometry in spleens (SPL) of the control (CTR) and TCL1 leukemia-bearing mice at the indicated stages of disease development (TCL1 early, TCL1 late). The graph shows results from two independent experiments; each dot represents an individual mouse, n = 5–14, Mann‒Whitney U test, ****p ≤ 0.0001. **c** Representative dot plots showing the percentage of Ly6C^+^ Ly6G^+^ cells in the spleens of the control (SPL) and TCL1 leukemia-bearing mice at the indicated stages of disease development (TCL1 early, TCL1 late) assessed by flow cytometry. **d** The geometric mean fluorescence intensity of the oxidized CM-H_2_-DCFDA probe in splenic CD11b^+^ Ly6G^+^ living cells of the control (CTR) and TCL1 leukemia-bearing mice at the indicated stages of disease development (TCL1 early, TCL1 late) analyzed by flow cytometry. The graph shows results from two independent experiments; each dot represents an individual mouse, n = 5–14, Mann‒Whitney U test, **p ≤ 0.01, ***p ≤ 0.001 (left panel). Representative histograms displaying CM-H_2_-DCFDA fluorescence intensity versus the number of CD11b^+^ Ly6G^+^ cells in the spleens of the control (SPL) and TCL1 leukemia-bearing mice at the indicated stages of disease development (TCL1 early, TCL1 late) assessed by flow cytometry (right panel). **e** Graph showing the percentage of granulocytes (Ly6G^+^) isolated from the spleens of the control (CTR) and TCL1 leukemia-bearing mice at the early stage of the disease (TCL1) that phagocytosed *Escherichia coli* bioparticles. The data are presented as the mean ± SD, n = 3–4, Mann‒Whitney U test * p ≤ 0.05; **f** The percentage of CD63^+^ granulocytes (Ly6G^+^) isolated from the spleens of the control (CTR) and TCL1 leukemia-bearing mice at the early stage of the disease (TCL1). The graph shows the results for cells stimulated with C5a at different concentrations (10 µM and 100 µM) as a fold of that of the unstimulated control cells, n = 3–7, Mann‒Whitney U test, *p ≤ 0.05, ***p ≤ 0.001, ****p ≤ 0.0001

In our experiments, we performed the adoptive transfer of TCL1 leukemic B cells isolated from the spleen of Eµ-TCL1 mouse into C57BL6/J wild-type (WT) mice, which led to the formation of a tumor immunosuppressive microenvironment and disease progression in the recipient animals [21]. To evaluate the phenotype of neutrophils, we collected the spleens (SPL) of TCL1 leukemia-bearing mice at two stages of disease development: early (4–10% of leukemic CD5^+^ CD19^+^ cells detected in the blood) and late (50–80% of leukemic CD5^+^ CD19^+^ cells detected in the blood) (Fig. 1A). Compared to that of the control WT animals (CTR), the percentage of splenic granulocytes among CD11b^+^ cells in TCL1 leukemia-bearing mice (TCL1) was significantly decreased (Fig. 1B, C). Moreover, the drop in the percentage of Ly6G^+^ cells seems to be dependent on leukemia progression, as the observed effect was much stronger for the TCL1 mice at the advanced stage of the disease. Regardless of the stage of leukemia, CLL neutrophils had an impaired ability to produce reactive oxygen species (ROS), as they presented exceptionally low fluorescence of DCFDA, the hydrogen peroxide indicator (Fig. 1D).

The disturbed ROS production in neutrophils from TCL1 leukemia-bearing mice prompted us to verify the other parameters of neutrophil functionality, such as phagocytosis and degranulation. To this end, we collected neutrophils from spleens only at the early stage of leukemia since the advanced leukemia burden made the Ly6G^+^ population too rare for effective separation. First, we applied flow cytometry to analyze the percentage of neutrophils phagocytosing *E. coli* bioparticles. The results indicated that leukemia-associated neutrophils phagocytosed *E. coli* bioparticles slightly less effectively than control neutrophils. Next, we evaluated the degranulation of neutrophil azurophilic granules by analyzing the expression of the surrogate marker CD63 on the plasma membrane. After the cells were primed with granulocyte macrophage-colony stimulating factor (GM-CSF), cytochalasin B was added to enhance the depolymerization of actin, and cells were activated with C5a. Flow cytometry analysis revealed an elevated percentage of CD63^+^ cells, which may suggest an increased degranulating potential of leukemic granulocytes when compared with cells isolated from healthy control mice (Fig. 1E, F).

**Fig. 2:**
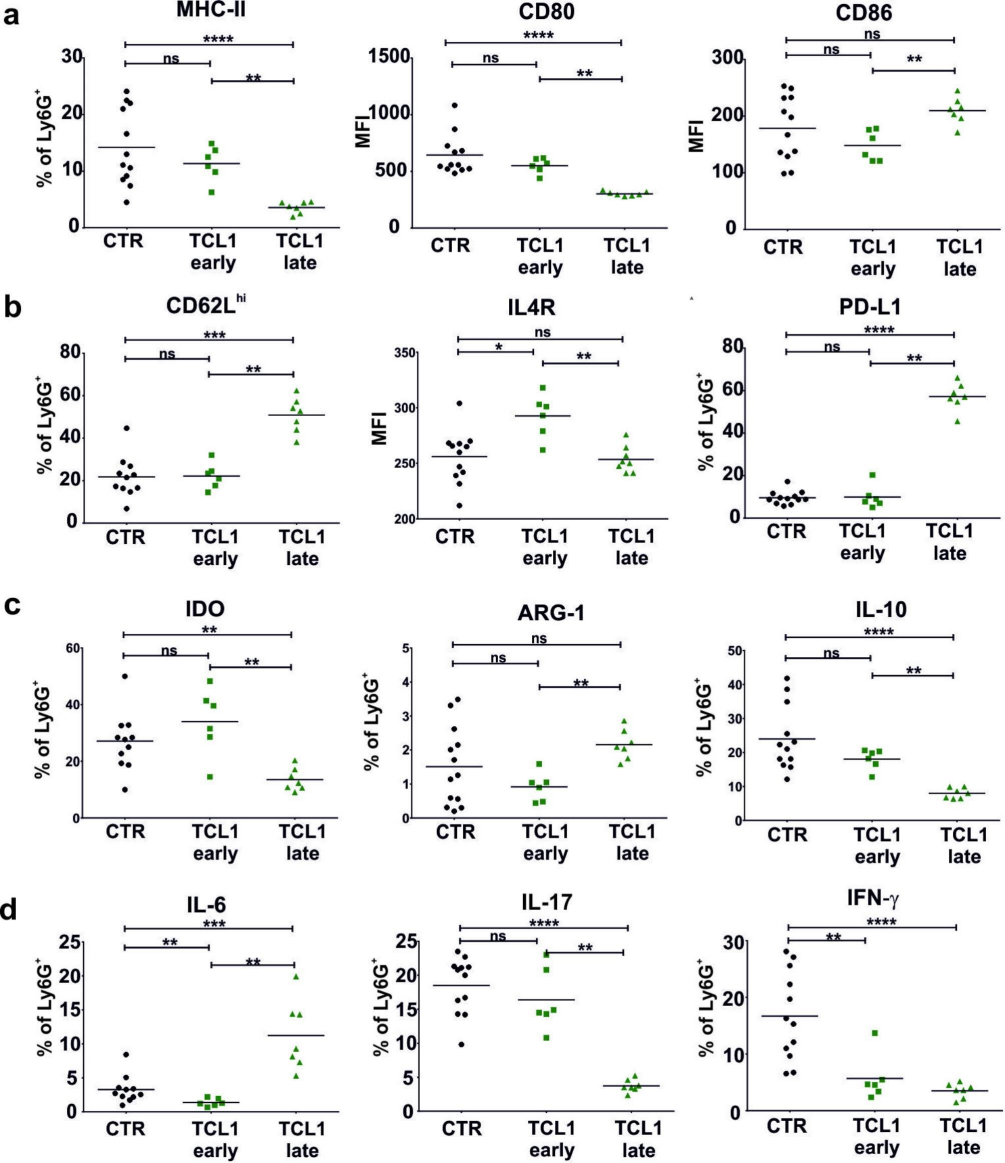
Phenotype of neutrophils in TCL1 leukemia-bearing mice. **a, b, c, d** The expression of selected surface (**a, b**) and intracellular (**c, d**) markers of granulocytes (CD11b^+^ Ly6G^+^) in spleens isolated from the control (CTR) and TCL1 leukemia-bearing mice at the indicated stages of disease development (TCL1 early – 4–10% of leukemic cells in blood, TCL1 late – 50–80% of leukemic cells in blood) assessed by flow cytometry. The graphs show results from two independent experiments; each dot represents an individual mouse, n = 7–12, Mann‒Whitney U test, ns – not significant, *p ≤ 0.05, **p ≤ 0.01, ***p ≤ 0.001, ****p ≤ 0.0001

Next, we examined the phenotype of leukemia-associated neutrophils at both the early and late stages of the disease. The results indicate that neutrophils expressing MHC-II and CD80 are less frequent in the TCL1 leukemia-bearing mice than in the control mice, and this phenomenon worsens with the progression of the disease (Fig. 2A, Supp. Figures 1 and 2). As reported previously for other myeloid cell populations in CLL [33], the percentage of leukemia-associated neutrophils expressing CD86 is increased at the advanced stage of leukemia. The progression of the disease was also correlated with the expansion of CD62L^high^ neutrophils. Additionally, we analyzed the expression of immunosuppressive factors in leukemia-associated neutrophils. PD-L1-positive neutrophils were more frequent in the late stage of disease than in the other studied groups. Additionally, the late stage of disease was associated with a higher percentage of neutrophils expressing ARG-1 than the early stage of leukemia (Fig. 2B C). Other populations expressing markers of immunosuppressive potential, such as IDO and IL4R, were more frequent at the early stage of leukemia. Subsequently, we evaluated the levels of cytokines in leukemia-associated neutrophils. The results indicated that the production of IL-10, IL-17 and IFN-γ is decreased and seems to be related to disease progression. In contrast, IL-6 production was elevated, and these effects were restricted only to neutrophils at the late stage of leukemia (Fig. 2D). The obtained results indicated that the neutrophils isolated from spleens (sites of proliferation of leukemic cells) show altered phenotype and function, and at least the phenotype depends on the stage of disease progression. Nevertheless, it is not clear whether these changes occur under the influence of the leukemia microenvironment or arise during the maturation of the cells.

### The CLL microenvironment triggers changes in the neutrophil phenotype

**Fig. 3:**
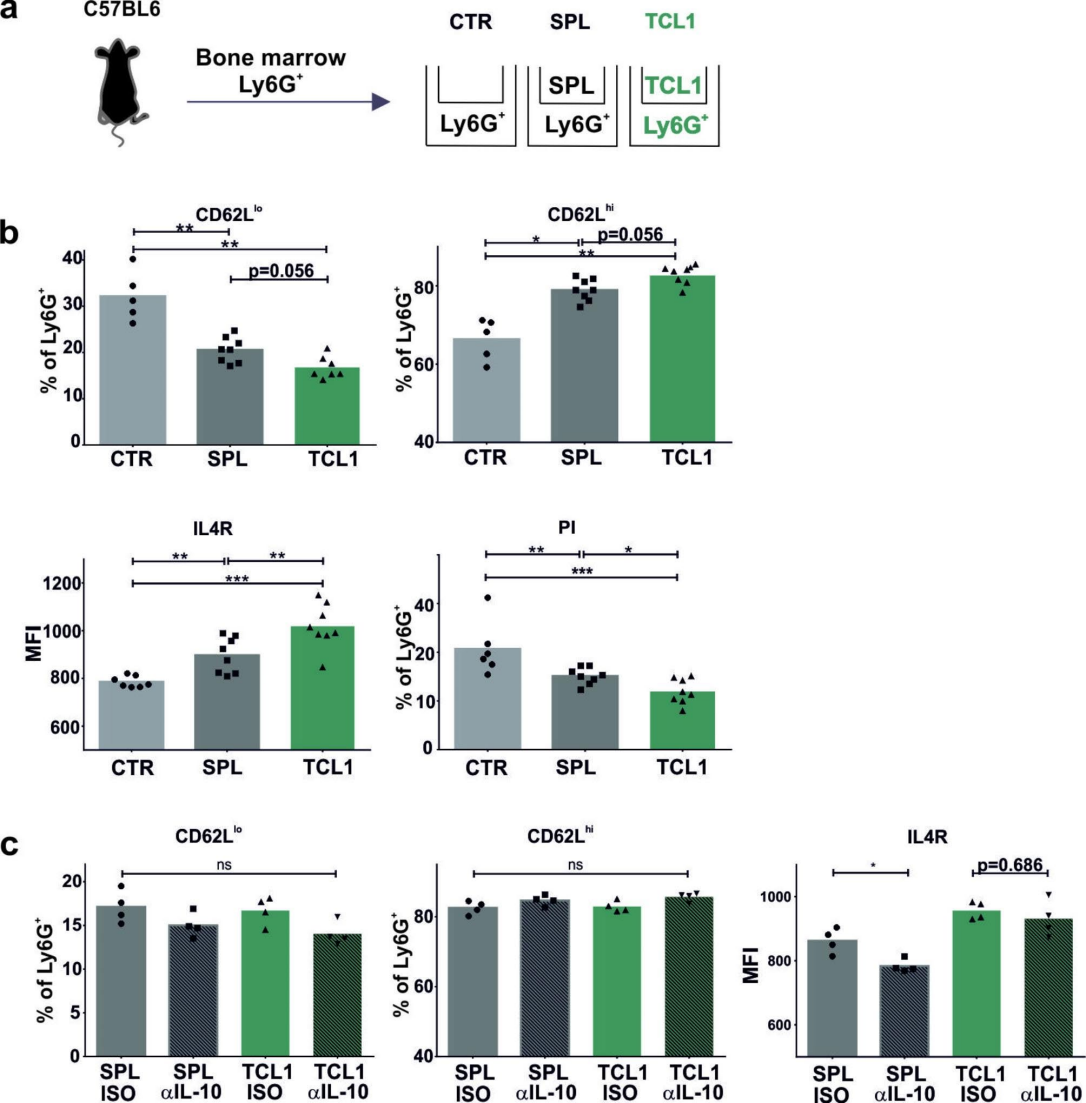
The influence of the leukemia microenvironment on the neutrophil phenotype. **a** Scheme of the experiment. Bone marrow Ly6G^+^ cells isolated from C57BL6/J WT donors were cocultured ex vivo alone (CTR) with splenocytes isolated from healthy donors (SPL) or from TCL1 leukemia-bearing mice (TCL1) at a 1:2 ratio for 19 h. Neutrophils and splenocytes were separated by Transwells to avoid direct cell‒cell interactions. **b** The percentage of bone marrow Ly6G^+^ cells with low (CD62L^lo^) and high (CD62L^hi^) expression of the CD62L surface marker upon incubation with no other cells (CTR), with splenocytes isolated from the healthy donors (SPL) or from TCL1 leukemia-bearing mice (TCL1) assessed by flow cytometry (upper panels); the expression of the IL4R surface marker on the bone marrow Ly6G^+^ upon incubation with no other cells (CTR), with splenocytes isolated from healthy donors (SPL) or from TCL1 leukemia-bearing mice (TCL1) assessed by flow cytometry (lower left panel, MFI – mean fluorescence intensity); the percentage of propidium iodide (PI)-positive bone marrow Ly6G^+^ cells upon incubation with no other cells (CTR), with splenocytes isolated from healthy donors (SPL) or from TCL1 leukemia-bearing mice (TCL1) assessed by flow cytometry (lower right panel). The graphs present both the value for each individual sample (points) and the mean value (bars), n = 5–8, Mann‒Whitney U test, *p ≤ 0.05, **p ≤ 0.01, ***p ≤ 0.001. **c** The bone marrow Ly6G^+^ cells were incubated with splenocytes isolated from healthy donors (SPL) or from TCL1 leukemia-bearing mice (TCL1) with the presence of isotype antibody (ISO) or anti-IL-10 antibody (αIL-10) added to the culture medium in a final concentration of 10 µg/ml for 19 h. The graphs show the expression of the CD62L and IL4R surface markers assessed by flow cytometry. The results are presented as both the value for each individual sample (points) and the mean value (bars), n = 4, Mann‒Whitney U test, ns – not significant, *p ≤ 0.05

The plasticity of neutrophils in the tumor microenvironment has been widely discussed and is characteristic of leukemia-associated neutrophils that exhibit an immunosuppressive phenotype. Although the majority of studies concerning phenotypes were performed on cells isolated from peripheral blood, it is still not clear how this immunosuppressive phenotype is induced. Therefore, in the next step, we investigated whether CD62L^high^ neutrophils might be generated in the tumor microenvironment or must migrate from bone marrow as immature cells. Neutrophils isolated from the bone marrow of healthy mice were cocultured with control splenocytes (SPL) or splenocytes isolated from TCL1 leukemia-bearing mice (TCL1) (Fig. 3A). Importantly, coculture with Transwells eliminated direct cell‒cell contact, enabling only the migration of secreted proteins (e.g., cytokines) and other chemical factors via the culture medium. Interestingly, coculture of both healthy SPL and TCL1-SPL led to an increased percentage of CD62L^high^ neutrophils; however, the effect did not reach statistical significance. Nevertheless, only factors produced by TCL1-SPL caused an increase in IL4R expression and improved the survival of neutrophils (Fig. 3B). The results obtained from ex vivo experiments suggest that the leukemia microenvironment may initiate a change in the neutrophil phenotype. Since IL-10 is secreted by leukemic B and Treg cells in the TCL1 model, we next investigated whether this cytokine plays a role in the change in neutrophil phenotype. To this end, we used antibodies (Abs) directed against IL-10 or a proper isotype control and added them to the coculture experiments. Neutralization of IL-10 did not affect the expression of CD62L; however, anti-IL-10 Abs decreased IL4R expression levels on neutrophils but only in the group cocultured with healthy SPL (Fig. 3C). These experiments revealed that even brief co-incubation of neutrophils with TCL1 leukemia cells altered the expression of CD62L and IL4R. In this experimental setup, however, the observed changes do not depend on IL-10, which is produced mainly by the leukemic cells.

### Induction of the antileukemia immune response restores the phenotype of neutrophils

**Fig. 4:**
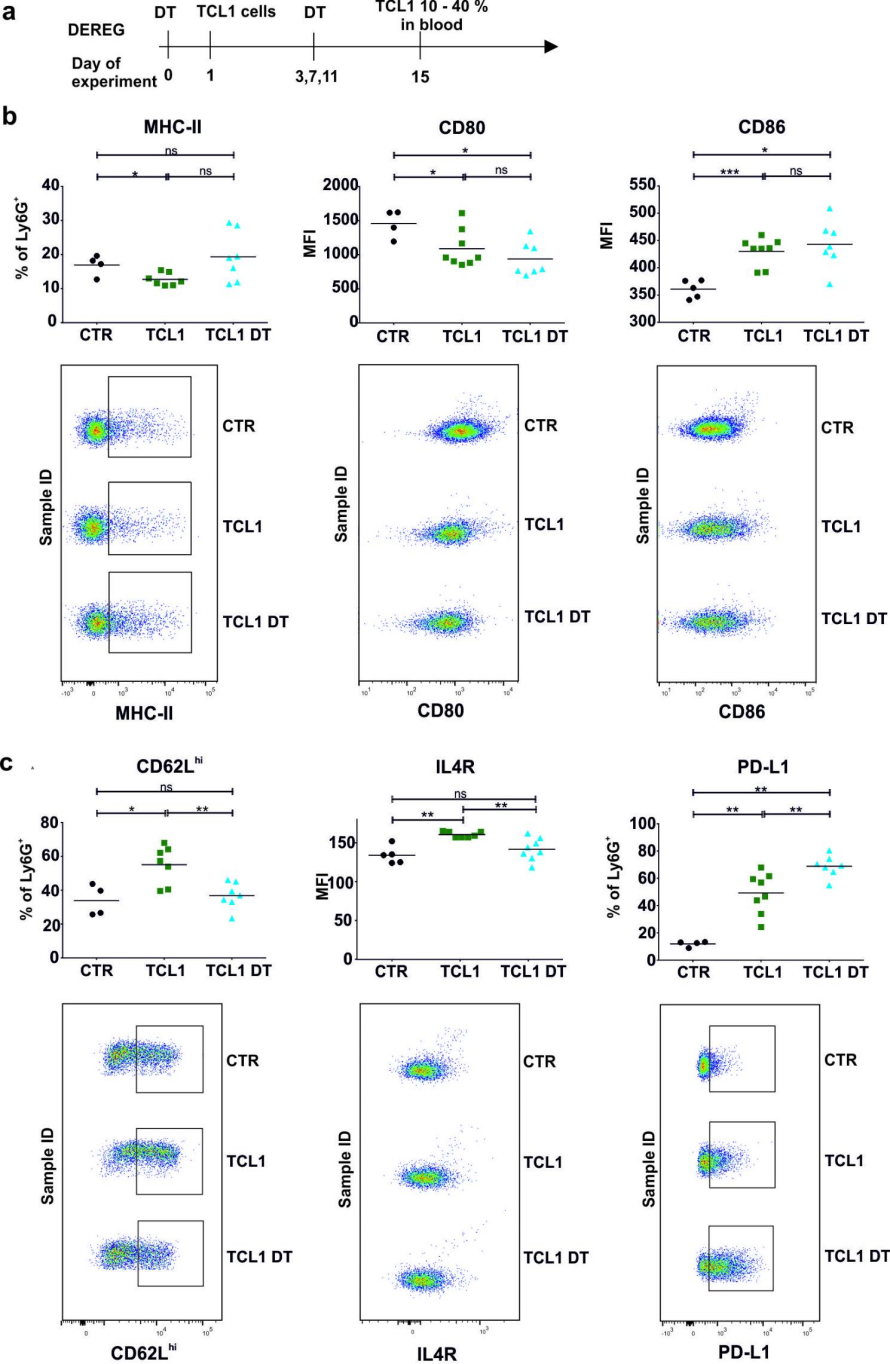
Phenotype of neutrophils in TCL1 leukemia-bearing mice upon induction of the antileukemic immune response by Treg depletion. **a** Scheme presenting the timeline of the experiment. Regulatory T cells (Treg) were depleted in DEREG mice using diphtheria toxin (DT) one day before TCL1 cells (CD5^+^ CD19^+^) injection. DT administration was repeated every 4 days. The phenotype of splenic neutrophils was analyzed by flow cytometry two weeks after leukemic cell inoculation, when there were 10–40% leukemic (CD5^+^ CD19^+^) cells among WBC in the blood. **b, c** The expression of the selected surface markers of splenic granulocytes (CD11b^+^ Ly6G^+^) isolated from the control (CTR) and TCL1 leukemia-bearing DEREG mice untreated (TCL1) or treated with DT (TCL1 DT), MFI – mean fluorescence intensity; the graphs presented in the upper panels show results from two independent experiments; each point represents an individual mouse, n = 4–7, Mann‒Whitney U test, ns – not significant, *p ≤ 0.05, **p ≤ 0.01, ***p ≤ 0.001. Representative dot plots are shown in the lower panels

Next, we investigated whether the induction of an antileukemic immune response may restore the “healthy/normal” neutrophil phenotype. Previously, we showed that depletion of Treg leads to leukemia eradication in TCL1 leukemia-bearing mice [22]. Therefore, to modify the immunosuppressive environment in leukemic spleens, we depleted Treg using diphtheria toxin (DT) in TCL1-injected DEREG mice. The phenotype of neutrophils was analyzed two weeks after TCL1 cells inoculation (Fig. 4A). At that time, the antileukemic immune response had already developed but had not yet eradicated leukemia. Flow cytometry analysis indicated that depletion of Treg led to slightly elevated MHC-II expression on neutrophils; however, the change was not significant (Fig. 4B). Treg depletion did not affect the expression of CD80 and CD86 but led to a decrease in the percentage of CD62L^high^ neutrophils to the level observed in the control mice (Fig. 4B, C).

Moreover, DT injection led to reduced expression of IL4R. Interestingly, the depletion of Treg caused an additional increase in PD-L1 expression. However, depletion of Treg did not affect the production of IL-6, IFN-γ, or IL-10 (Supp. Figure 3). Additionally, we analyzed the correlation between the expression of markers used for phenotyping and leukemia progression (Supp. Tables 2 and Supp. Figure 4). When the percentage of leukemic cells in the animals’ blood was analyzed, the correlation reached statistical significance for MHC-II and CD80. In contrast, the progression of leukemia into the spleen correlates with the expression of MHC-II, CD62L, IL4R and PD-L1. The obtained results suggest that the neutrophil phenotype is affected by both the presence of leukemic cells and Treg. This effect is more clearly related to the percentage of leukemic cells in the spleen, which also changes under the influence of activation of the immune system due to Treg depletion [22].

**Fig. 5:**
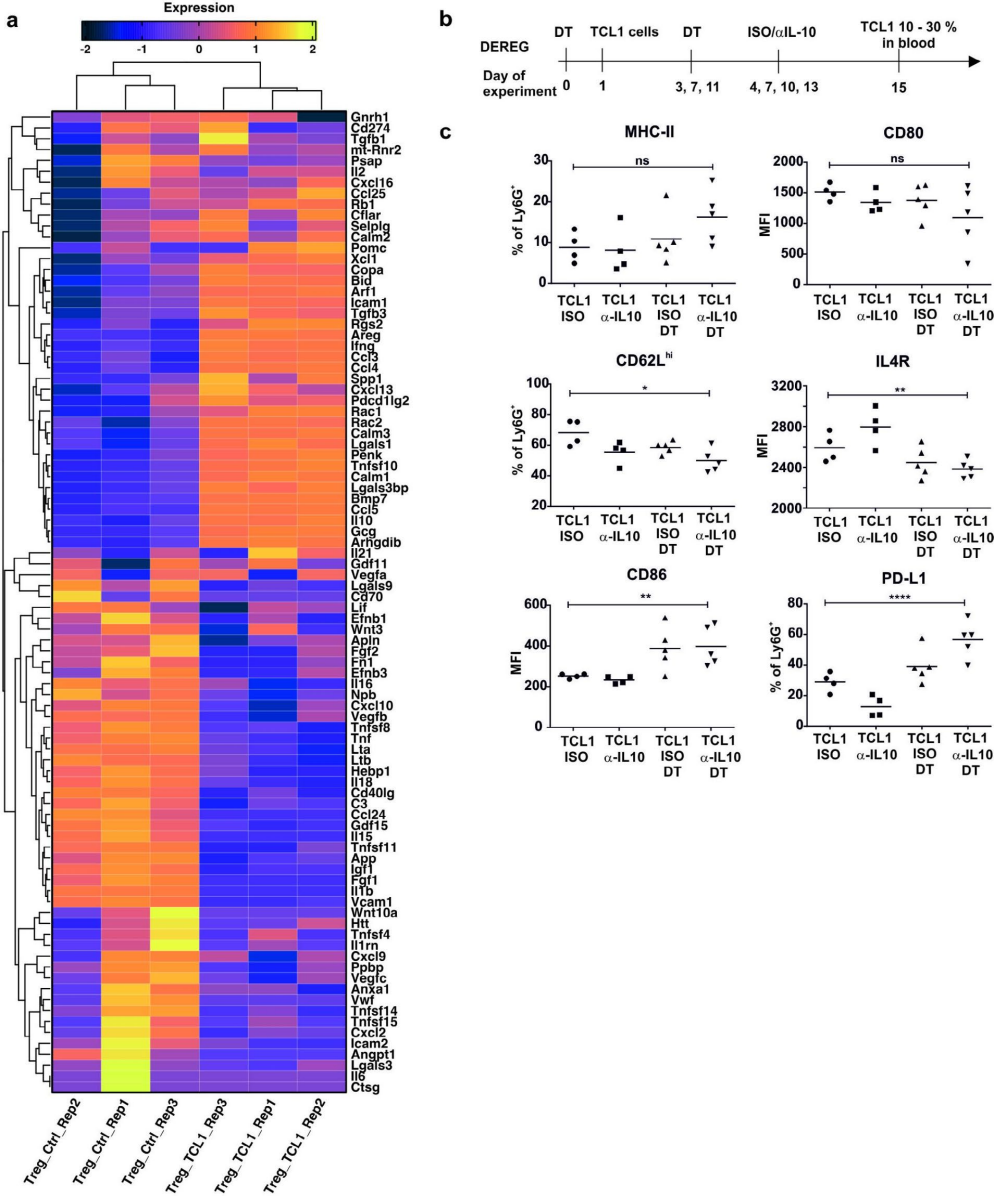
The Treg and IL-10 in shaping the neutrophil phenotype. **a** Clustering of selected differentially expressed genes encoding receptor ligands in Treg isolated from control (Treg_Ctrl) and TCL1 leukemia-bearing mice (Treg_TCL1) 14 days after TCL1 leukemia (CD5^+^ CD19^+^) cell inoculation, when there were 10–30% leukemic cells among WBC in the blood. **b** Scheme presenting the timeline of the experiment. Treg were depleted in DEREG mice using diphtheria toxin (DT) one day before TCL1 cells injection. DT administration was repeated every 4 days. Anti-IL10 antibody (α-IL10) or the appropriate isotype control (ISO) were administered every 3 days starting from Day 4. The phenotype of splenic neutrophils was analyzed by flow cytometry two weeks after TCL1 leukemia (CD5^+^ CD19^+^) cells inoculation, when there were 10–30% leukemic cells among WBC in the blood. **c** The expression of the selected surface markers of splenic granulocytes (CD11b^+^ Ly6G^+^) isolated from TCL1 leukemia-bearing DEREG mice untreated (TCL1) or treated with DT (TCL1 DT) and treated with anti-IL10 antibody (α-IL10) or isotype control (ISO), MFI – mean fluorescence intensity; each point represents an individual mouse, n = 4–5, Mann‒Whitney U test, ns – not significant, *p ≤ 0.05, **p ≤ 0.01, ****p ≤ 0.0001

Notably, in our previous work, we found a leukemia-specific subpopulation of Treg, defined as CD44^lo^CD25^lo^LAG3^+^ [22]. This newly described subpopulation of Treg can be a source of cytokines that modify neutrophil function. Thus, we reanalyzed the previously collected RNA-seq data from TCL1 Treg and control Treg cells, screening for factors potentially important for neutrophil functions and phenotypes (Fig. 5A, Supp. Tables 3 and 4). The obtained results indicate that leukemic Treg express significantly more Il10, Ifnγ, and chemokines (Ccl3/4/5, Cxcl13), which can all contribute to the immunosuppressive phenotype of neutrophils. Altogether, our data suggest that depletion of Treg in the TCL1 model induces changes in the neutrophil phenotype by creating a more antileukemic microenvironment and limiting the availability of immunosuppressive cytokines.

Since the immunosuppressive effect of IL-10 on neutrophils has been widely discussed, we investigated whether the neutralization of IL-10 may additionally contribute to the change in neutrophil phenotype obtained by Treg depletion. We observed that depletion of Treg and administration of anti-IL-10 Abs elevated the level of MHC-II expression on neutrophils, however, this effect was not statistically significant (Fig. 5B, C). Still, the IL-10 neutralization did not affect CD80 or CD86 expression on the neutrophil surface when compared to that of the cells isolated from mice receiving DT alone. The percentage of CD62L^high^ neutrophils was significantly reduced by anti-IL-10 Abs and Treg depletion alone as well as the combination of both when compared to that of the control mice (receiving only isotype control Abs). Importantly, our results indicate that the elimination of Treg was crucial for the reduction in IL4R expression on neutrophils; however, coadministration of anti-IL10 Abs did not influence this effect. Nevertheless, the combination of Treg depletion with anti-IL-10 Abs resulted in a significant increase in PD-L1 expression in comparison to neutrophils obtained from control mice (Fig. 5C). Although Treg depletion causes changes in the neutrophil phenotype, it is not clear whether they are the result of the lack of a Treg population or secondary changes due to activation of the immune system.

### Treg induce the expression of IL4R in neutrophils of RAG2KO mice

**Fig. 6:**
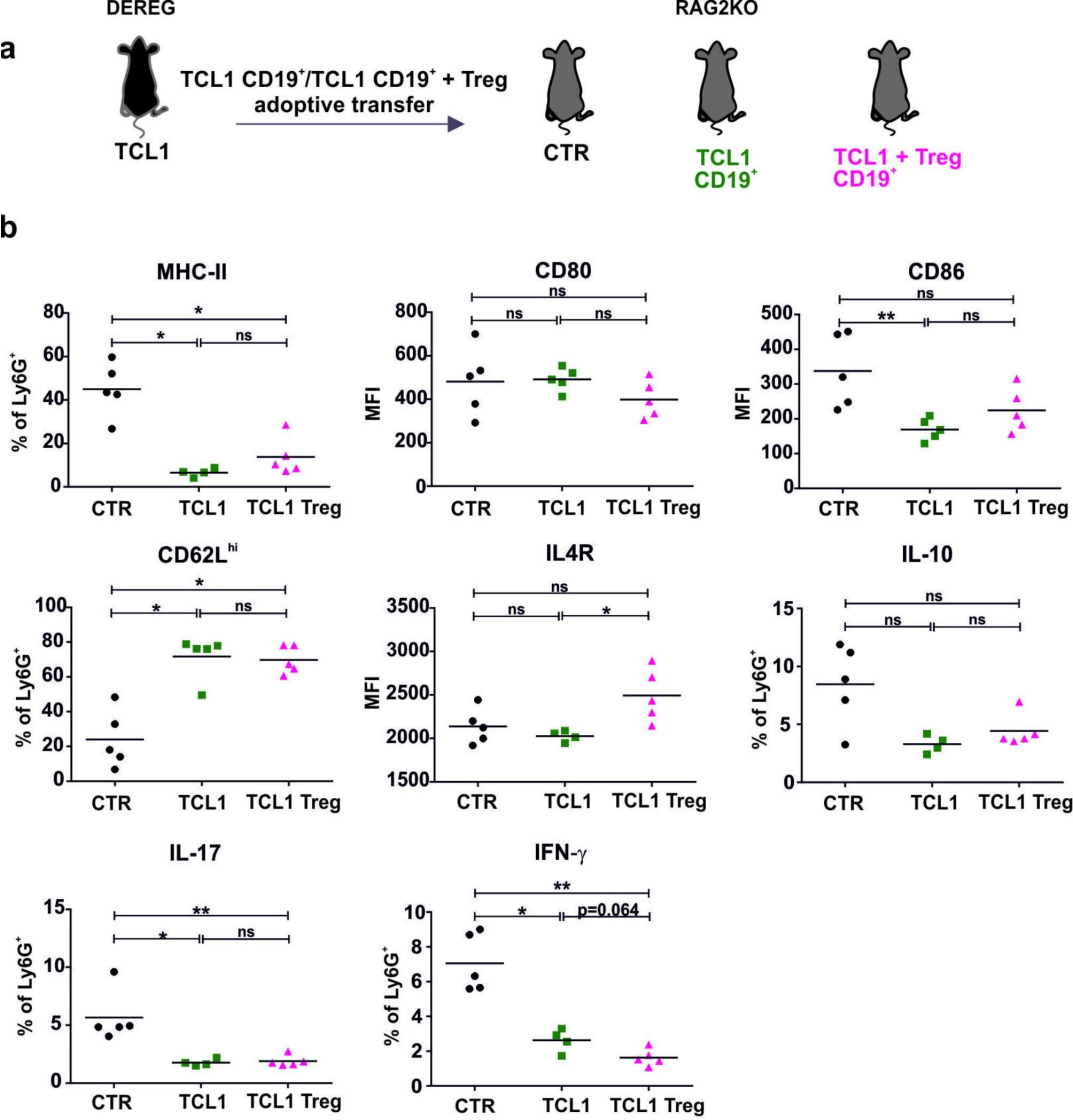
The influence of Treg on the neutrophil phenotype. **a** Scheme of the experiment. RAG2KO mice were inoculated with TCL1 leukemic cells alone (TCL1 CD19^+^) or together with GFP + Treg (TCL1 CD19^+^ + Treg) sorted from the spleens of TCL1 leukemia-bearing DEREG mice. Upon 28 days, the animals were sacrificed, and splenocytes were isolated to analyze the phenotype of granulocytes by using flow cytometry. **b** The expression of the selected markers of granulocytes (CD11b^+^ Ly6G^+^) isolated from the spleens of RAG2KO mice with no leukemia (CTR) and upon adoptive transfer of TCL1 leukemia cells alone (TCL1 CD19^+^) or together with GFP^+^ Treg (TCL1 CD19^+^ + Treg), MFI – mean fluorescence intensity; each point represents an individual mouse, n = 4–5, Mann‒Whitney U test, ns – not significant, *p ≤ 0.05, **p ≤ 0.01

To further investigate the influence of Treg on the neutrophil phenotype and to better recreate the microenvironmental conditions instead of difficult-to-maintain in vitro cocultures, we conducted experiments in the RAG2KO model. To this end, RAG2KO mice were injected with leukemic cells (CD19^+^) alone or together with GFP^+^ Treg sorted from TCL1 leukemia-bearing DEREG mice (Fig. 6A). Adoptive transfer of Treg did not affect the level of MHC-II, CD80 or CD86 expression on splenic neutrophils in these mice. However, the results obtained from this experiment revealed that Treg are responsible for the increase in IL4R expression on leukemia-associated neutrophils (Fig. 6B). In these experimental settings, we were also able to detect a high percentage of CD62L^high^ neutrophils in TCL1 leukemia-bearing mice; however, adoptive transfer of Treg did not change this effect. Additionally, neutrophils from the mice inoculated with TCL1 leukemia cells produced IL-10, IL-17, and IFN-γ at lower levels than neutrophils from the control mice, and adoptively transferred Treg did not reverse this outcome (Fig. 6B, D).

## Discussion

Neutrophils are important players in cancer-related inflammation and contribute to tumor spread and disease progression. The role of neutrophils in the progression of CLL has already been underlined by Gatjen et al. The depletion of Ly6G^+^ cells delayed leukemia cell proliferation in the mouse Eµ-TCL1 model [14]. In these studies, neutrophils isolated from the bone marrow showed no changes in ROS production or in phenotype [12, 14]. Some differences were observed in cells isolated from blood or spleens, where they establish the leukemia microenvironment. On this basis, we adopted the assumptions of our work to study the phenotype and function of neutrophils in the spleen – the site where the crosstalk between neutrophils and leukemic cells occurs. Nevertheless, we did not deplete the Ly6G^+^ cells since the results showing the depletion of populations may raise some controversies. Recently, Boivin et al. showed that neutrophils remaining after anti-Ly6G treatment are newly derived from the bone marrow, and these new neutrophils have lower Ly6G membrane expression, leading to a diminution in the target level for anti-Ly6G Abs [34]. To date, numerous studies have highlighted the plasticity of neutrophils in different pathological states [35–37] and described various approaches enabling the reprogramming of neutrophil functions [38–40]. Importantly, the ability of tumor cells to change neutrophil phenotypes and functions has been broadly reported in studies on the solid tumor microenvironment. Nevertheless, the protumor phenotype of neutrophils is not fully defined, as these cells are challenging to study due to their short lifespan and the inability to genetically manipulate, cryopreserve or expand them in vitro [41]. Moreover, the availability of data concerning neutrophils in hematological diseases is limited. Therefore, in this study, we aimed to define the neutrophil phenotype of TCL1 leukemia-bearing mice isolated from the leukemia microenvironment. In the TCL1 mouse model, similar to human CLL, we observed a lower number of neutrophils, which is associated with disease progression [6]. It was already reported by Manukyan et al. that human neutrophils of CLL patients are activated and reveal functional defects that can be related to the clinical course of diseases [13]. In our studies, neutrophils from TCL1 leukemia-bearing mice also revealed alterations, as we detected a significant decrease in the percentage of MHC-II- and CD80-expressing neutrophils. However, our data showed that the frequencies of CD86-expressing neutrophils decreased at the early stage and increased in the late stage of the disease. The CD86 marker on neutrophils may be modulated by various inflammatory factors, such as IL-4 and TNF-α, which are induced in CLL patients [42, 43]. Moreover, in contrast to human CLL blood neutrophils, among mouse leukemia-associated neutrophils, we observed high frequencies of CD62L-expressing cells at the late stage of disease [13]. According to previous studies, CD62L is cleaved from the cell surface during priming or, as presented by Tak et al. [44], the low expression of CD62L may be associated with a neutrophil subset called “aged”, which is rapidly recruited to the bloodstream in response to acute inflammation [45]. The results obtained in our study concerning a high percentage of CD62L^high^-expressing neutrophils indicate that mouse leukemia-associated neutrophils lost their activity potential, and this effect may be mediated by the factors released by the leukemia microenvironment. Moreover, experiments with TCL1 leukemia-bearing DEREG mice show that Treg depletion reduces the frequencies of CD62L^high^ expressing cells (meaning increased activation) and induces an antileukemia immune response. Moreover, as previously published by others, Treg can inhibit neutrophil activation and accumulation [46, 47]. Given the changes we observed in the neutrophil phenotype and the data showing loss of function by neutrophils in CLL patients [13, 48], a high level of CD62L might be a marker of mouse CLL-associated neutrophil loss of proinflammatory functions. According to our results, mouse leukemia-associated neutrophils exhibit functional defects that manifest in their limited ROS production, decreased capability of phagocytosis, and reduced cytokine production. These results are in agreement with a previously mentioned study by Manukyan et al., who reported functional defects in the circulating neutrophils of CLL patients [13]. In fact, our data also revealed that the level of azurophilic degranulation was elevated in leukemia-associated neutrophils. Usually, the process of degranulation is tightly controlled; however, dysregulation of content granule release has been reported in cancer diseases. A high level of granule content may be beneficial for tumor development and progression, as shown for many types of solid tumors and acute promyelocytic leukemia [49]. We assume that the described phenotype provides evidence that TCL1 leukemia-associated neutrophils were polarized into leukemia-supporting cells, similar to observations made by Gatjen et al. [14]. Moreover, our ex vivo results indicated that factors secreted by TCL1 leukemic cells lead to a decrease in the CD62L^low^ and an increase in the CD62L^high^ percentage in the control neutrophil population isolated from healthy animals. This finding may suggest that the CD62L^high^ neutrophils present in the leukemic spleens are likely modified by a tumor microenvironment.

Tumor-associated neutrophils were consistently reported to reveal immunosuppressive phenotypes. Therefore, we analyzed the neutrophil populations expressing surface molecules known for their immunosuppressive action. Interestingly, flow cytometry revealed that the percentage of TCL1 leukemia-associated neutrophils expressing PD-L1 and ARG-1 was increased at the late stage of disease, whereas the percentage of IDO- and IL4R-expressing cells was higher at the early stage. Our results are in line with the data published by Romano et al. showing that neutrophils in classic Hodgkin lymphoma are immunosuppressive through increased expression of ARG-1, which was higher in patients with advanced stage disease [50]. Our findings concerning PD-L1-expressing neutrophils are in accordance with currently available data. To date, it has been shown that in some types of solid tumors, increased frequencies of PD-L1-expressing neutrophils are present in the tumor microenvironment. These cells are associated with disease progression and reduced patient survival [51, 52]. The significant increase in PD-L1 expression in neutrophils after Treg depletion may be a consequence of the release of IFN-γ, the production of which is significantly enhanced as a result of the activation of the antitumor immune response [22]. Interestingly, IL-10 neutralization also increases PD-L1 expression but only after Treg depletion. These results are consistent with the observation that IL-10 suppression enhances antitumor T cell activity and may explain why the neutralization of IL-10 improves the sensitivity of the TCL1 model to anti-PD-L1 therapy [53].

Moreover, the results of this study are consistent with the data published by Öztürk et al. showing increased expression of IDO on the surface of leukemia-associated neutrophils in the mouse Eµ-TCL1 model [54]. Additionally, high expression of IL4Rα in leukemia-associated neutrophils was observed in our study. It was shown that IL-4, through IL4R signaling in neutrophils, suppresses their infiltration and antimicrobial functions [55] and protects neutrophils from apoptosis [56]. However, our results obtained from ex vivo experiments suggest that TCL1 leukemic cells induced the expression of IL4Rα on neutrophils, although the adoptive transfer of Treg, was needed to elevate neutrophil IL4Rα in RAG2KO mice in vivo. A previous report showed that Treg can modulate the migration and function of neutrophils through the production of CXCL8 and IL-10 and the modulation of suppressor of cytokine signaling 3 (SOCS 3) in neutrophils [57, 58]. RNA-seq analysis of the Treg subpopulation from the murine CLL model revealed its unique phenotype characterized by upregulation of multiple factors, including Il10, Ifnγ, and the chemokines Ccl3/4/5 Cxcl13, which likely contribute to the neutrophil leukemia-supporting phenotype. Furthermore, the modification of the tumor microenvironment by induction of the antileukemic immune response and depletion of Treg by DT in DEREG TCL1 leukemia-bearing mice were able to decrease the frequencies of IL4R-expressing neutrophils. Nevertheless, the depletion of IL-10 did not influence the leukemic phenotype of neutrophils in either in vitro or in vivo experiments. Our results also indicated that the production of all tested cytokines, apart from IL-6, was reduced in leukemia-associated neutrophils. This finding additionally highlights the dysfunctionality of neutrophils in CLL. The modification of the tumor microenvironment by induction of the antileukemic immune response and depletion of Treg were not efficient in changing the cytokine profile of leukemia-associated neutrophils.

Although neutrophils isolated from blood or bone marrow have already been studied in the context of the course of CLL, our research focused on the phenotype and function of neutrophils present in the leukemia microenvironment. The obtained results support current data by clearly showing the plasticity phenomenon of neutrophils. We conclude that the change into the immunosuppressive phenotype of neutrophils is induced by B leukemic lymphocytes and Treg, and can be modified again by Treg depletion and the induction of an antileukemic immune response. Interestingly, on the basis of the obtained results, it seems that apart from leukemic cells, Treg can significantly affect the phenotype and functions of neutrophils, which, however, requires further in-depth studies.

### Electronic supplementary material

Below is the link to the electronic supplementary material.

Supplementary Material 1

## Data Availability

RNA-seq data were deposited in the NCBI GEO database (GSE179121). The remaining data generated during the current study are available from the corresponding author upon reasonable request.

## Abbreviations

Abs: antibodies
APRIL: A proliferation-inducing ligand
ARG1: arginase 1
BAFF: B-cell-activating factor receptor
CLL: chronic lymphocytic leukemia
IDO: indoleamine 2,3-dioxygenase
IFN-β: interferon β
IL: interleukin
MMP9: matrix metallopeptidase 9
NETs: neutrophil extracellular traps
PROK2: prokineticin 2
SPL: splenocytes
TGF-β: transforming growth factor β
VEGF: vascular endothelial growth factor
