# Supplementary material for: Regulatory T cells contribute to the immunosuppressive phenotype of neutrophils in a mouse model of chronic lymphocytic leukemia
Source: Exp Hematol Oncol. 2023 Oct 10;12:89. doi: 10.1186/s40164-023-00452-9 (PMC10563345; doi:10.1186/s40164-023-00452-9)
Supplement: Supplementary file 1 — Supplementary Material 1 [file 40164_2023_452_MOESM1_ESM.docx]

**SUPPLEMENTARY INFORMATION**

**Regulatory T cells contribute to the immunosuppressive phenotype of neutrophils in a mouse model of chronic lymphocytic leukemia**

Agnieszka Goral^1^*, Marta Sledz^2,5^*, Aneta Manda-Handzlik^3^, Adrianna Cieloch^3,5^, Alicja Wojciechowska^2^, Mieszko Lachota^2,4^, Agnieszka Mroczek^3,5^, Urszula Demkow^3^, Radoslaw Zagozdzon^2^, Katarzyna Matusik^5^, Malgorzata Wachowska^3^, Angelika Muchowicz^1,2^#

^1^Department of Immunology, Medical University of Warsaw, 02-097 Warsaw, Poland

^2^Department of Clinical Immunology, Medical University of Warsaw, 02-097 Warsaw, Poland

^3^Department of Laboratory Diagnostics and Clinical Immunology of Developmental Age, Medical University of Warsaw, 02-091 Warsaw, Poland

^4^ Department of Ophthalmology, Children’s Memorial Health Institute, 04-730 Warsaw, Poland

^5^Doctoral School, Medical University of Warsaw, 02-091 Warsaw, Poland

* Contributed equally

# Correspondence: Angelika Muchowicz, [angelika.muchowicz@wum.edu.pl](mailto:angelika.muchowicz@wum.edu.pl);

| **ANTIBODY** | **SOURCE** | **IDENTIFIER** |
| --- | --- | --- |
| IFN gamma Monoclonal Antibody (XMG1.2), FITC, eBioscience™ | ThermoFisher Scientific/  eBioscience | Cat#: 11-7311-41  RRID:AB_10718840 |
| PE Rat Anti-Mouse IL-17A  Clone TC11-18H10 (RUO) | BD Biosciences | Cat#: 559502  RRID:AB_397256 |
| APC Hamster anti-Mouse CD80  Clone 16-10A1 (RUO) | BD Biosciences | Cat#: 560016  RRID:AB_1645212 |
| BV510 Rat Anti-Mouse I-A/I-E  Clone M5/114.15.2 (also known as M5/114) (RUO) | BD Biosciences | Cat#: 742893  RRID:AB_2741133 |
| PerCP-Cy™5.5 Rat Anti-Mouse CD19  Clone 1D3 (RUO) | BD Biosciences | Cat#: 551001  RRID:AB_394004 |
| PE-Cy™7 Rat Anti-Mouse CD4  Clone GK1.5 (RUO) | BD Biosciences | Cat#: 563933  RRID:AB_2738492 |
| PerCP-Cy™5.5 Rat Anti-CD11b  Clone M1/70 (RUO) | BD Biosciences | Cat#: 550993  RRID:AB_394002 |
| FITC Rat Anti-CD11b  Clone M1/70 (RUO) | BD Biosciences | Cat#: 557396  RRID:AB_396679 |
| CD11b Monoclonal Antibody (M1/70), PE, eBioscience™ | ThermoFisher Scientific/  eBioscience | Cat#: 12-0112-81  RRID:AB_465546 |
| BV510 Rat Anti-Mouse CD62L  Clone MEL-14 (RUO) | BD Biosciences | Cat#: 563117  RRID:AB_2738013 |
| PerCP-Cy™5.5 Rat Anti-Mouse Ly-6C  Clone AL-21 (RUO) | BD Biosciences | Cat#: 560525 RRID:AB_1727558 |
| V450 Rat Anti-Mouse Ly-6C  Clone AL-21 (RUO) | BD Biosciences | Cat#: 560594  RRID:AB_1727559 |
| APC-Cy™7 Rat Anti-Mouse Ly-6G  Clone 1A8 (RUO) | BD Biosciences | Cat#: 560600  RRID:AB_1727561 |
| IDO Monoclonal Antibody (mIDO-48), eFluor 660, eBioscience™ | ThermoFisher Scientific/  eBioscience | Cat#: 50-9473-82  RRID:AB_2574335 |
| PE Rat Anti-Mouse CD124  Clone mIL4R-M1 (RUO) | BD Biosciences | Cat#: 552509  RRID:AB_394407 |
| Brilliant Violet 421™ anti-mouse CD274 (B7-H1, PD-L1) Antibody, clone: 10F.9G2 | BioLegend | Cat#: 124315  RRID:AB_10897097 |
| APC Rat Anti-Mouse IL-10  Clone JES5-16E3 (RUO) | BD Biosciences | Cat#: 554468  RRID:AB_398558 |
| APC anti-mouse IL-6 Antibody  Clone MP5-20F3 (RUO) | BioLegend | Cat#: 504508  RRID:AB_10694868 |
| Human/Mouse Arginase 1/ARG1 PE-conjugated Antibody | R&D Systems | Cat#: IC5868P |
| BV421 Rat Anti-Mouse CD86  Clone GL1 (RUO) | BD Biosciences | Cat#: 564198  RRID:AB_2738663 |
| BD Pharmingen™ PE Rat Anti-Mouse CD63PE Rat Anti-Mouse CD63 | BD Biosciences | Cat#: 564222  RRID:AB_2738678 |

**Supp. Table 1.** Antibodies used for flow cytometry.

**
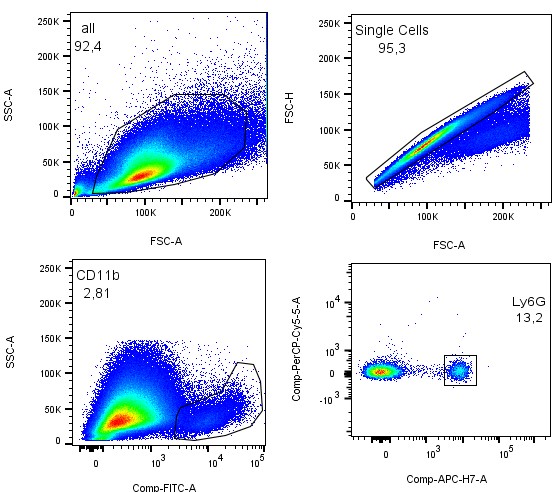
**

**Supp. Fig. 1.** Gating strategy for neutrophils in mice spleens.

**
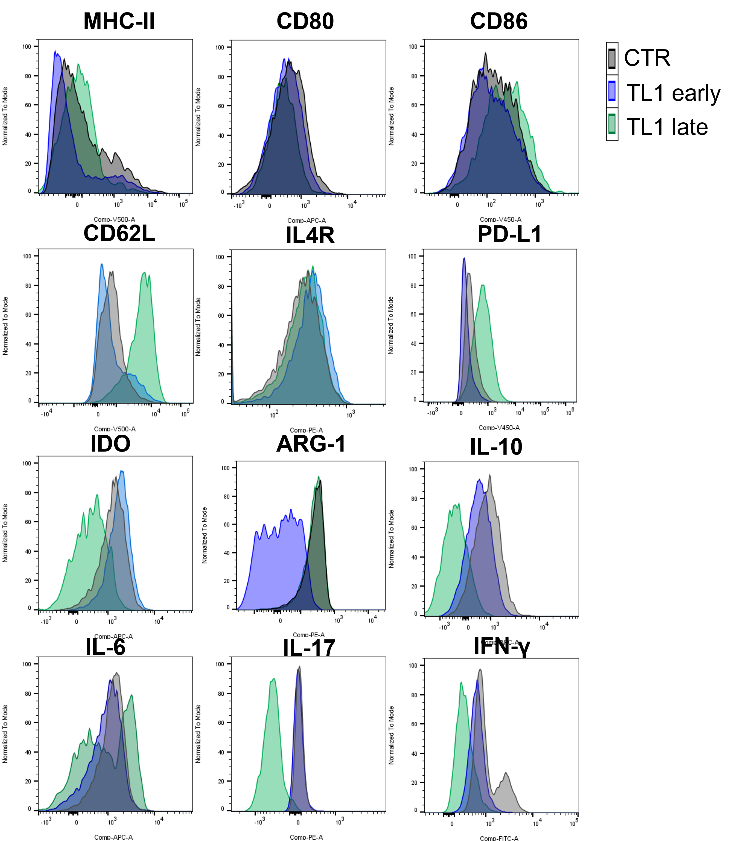
**

**Supp. Fig. 2.** Sample histograms show the level of expression of the markers described in Fig. 2.

**
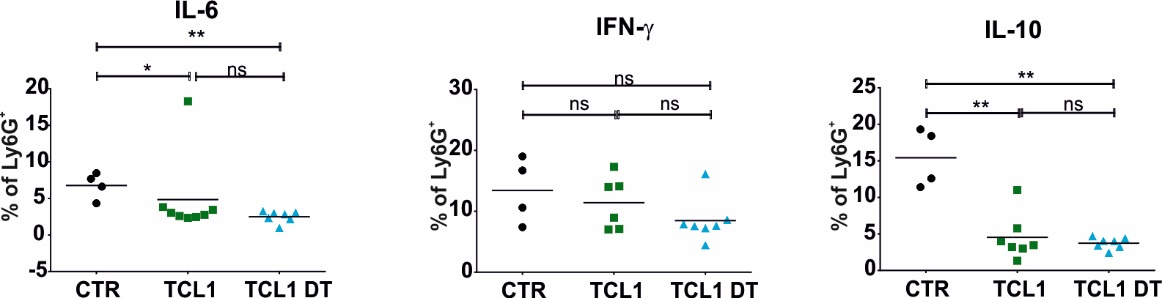
**

**Supp. Fig. 3.** The production of cytokines (IL-6, INF-γ and IL-10) by granulocytes (Ly6G^+^) from control (CTR) and TCL1 leukemia-bearing DEREG mice untreated (TCL1) or treated with DT (TCL1 DT). The graphs show results from two independent experiments; each point represents an individual mouse, n=4-7, Mann‒Whitney U test, ns – not significant, *p≤ 0.05, **p≤ 0.01.

| Sample  name | CD5+CD19+ (% ) | | MHC-II (%) | CD80 (MFI) | CD86 (MFI) | CD62Lhi (%) | IL4R (MFI) | PD-L1 (%) |
| --- | --- | --- | --- | --- | --- | --- | --- | --- |
|  | blood | spleen |  |  |  |  |  |  |
| CTR 1 | nd | nd | 18,2 | 1617 | 363 | 39,7 | 125 | 13,3 |
| CTR 2 | nd | nd | 17,2 | 1621 | 376 | 43,7 | 135 | 13 |
| CTR 3 | nd | nd | 19,6 | 1194 | 377 | 25,7 | 134 | 12,5 |
| CTR 4 | nd | nd | 12,7 | 1399 | 347 | 26,6 | 124 | 8,88 |
| TCL1 1 | 26 | 64 | 11,6 | 959 | 435 | 62,1 | 157 | 46,6 |
| TCL1 2 | 36 | 64 | 10,9 | 1166 | 392 | 64,2 | 159 | 61,6 |
| TCL1 3 | 23 | 75 | 15,4 | 1375 | 391 | 57,3 | 165 | 43,8 |
| TCL1 4 | 32 | 65 | 11 | 958 | 445 | 39,6 | 164 | 56,9 |
| TCL1 5 | 19 | 60 | 13 | 881 | 436 | 68 | 164 | 33,8 |
| TCL1 6 | 32 | 61 | 14,9 | 904 | 447 | 53,9 | 157 | 67,9 |
| TCL1 7 | 35 | 61 | 12,1 | 851 | 460 | 40,4 | 162 | 59,4 |
| DT 1 | 35 | 57 | 19,4 | 757 | 439 | 33 | 130 | 74,2 |
| DT 2 | 40 | 61 | 11,3 | 763 | 423 | 45 | 118 | 64,6 |
| DT 3 | 39 | 58 | 11,8 | 696 | 370 | 46 | 144 | 69,3 |
| DT 4 | 38 | 56 | 16 | 788 | 464 | 36,9 | 136 | 80,3 |
| DT 5 | 16 | 46 | 19 | 1097 | 429 | 39,3 | 149 | 54,9 |
| DT 6 | 15 | 39 | 29,3 | 1128 | 509 | 34,3 | 138 | 67,9 |
| DT 7 | 10 | 43 | 28,5 | 1342 | 468 | 23,3 | 156 | 70,5 |

**Supp. Table 2**. The percentage of leukemic cells (CD5^+^ CD19^+^) in blood and spleens of DEREG mice after Treg depletion. Table contains the percentage or MFI values in each animal used for graph preparation in Supp. Fig. 4.

**Supp. Fig. 4.** The correlation assessed based on Suppl. Table 2 data. Graphs present nonparametric Spearman correlation.


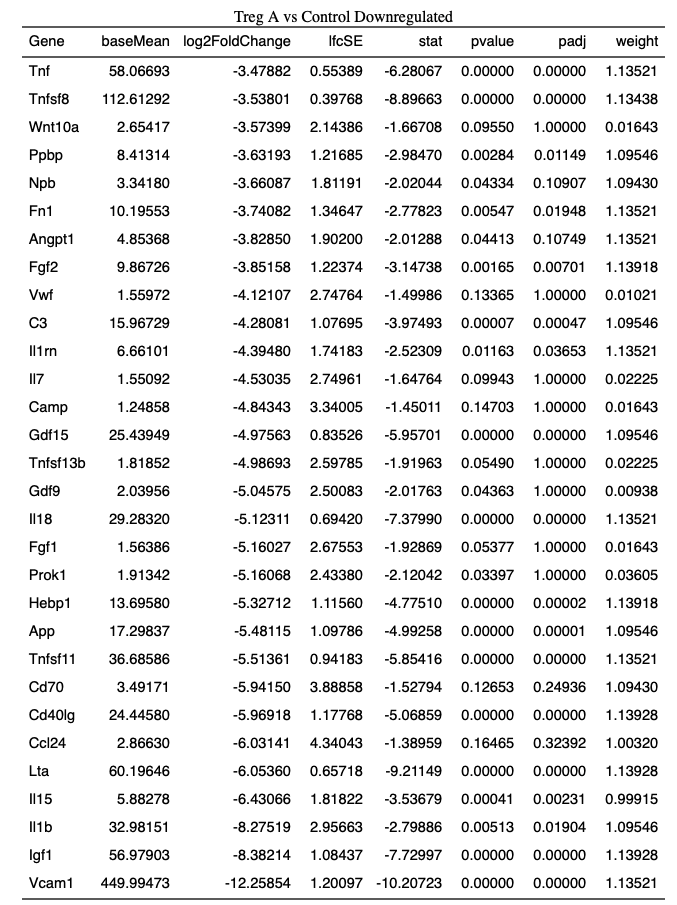


**Supp. Table 3**. The table with computed downregulated genes.


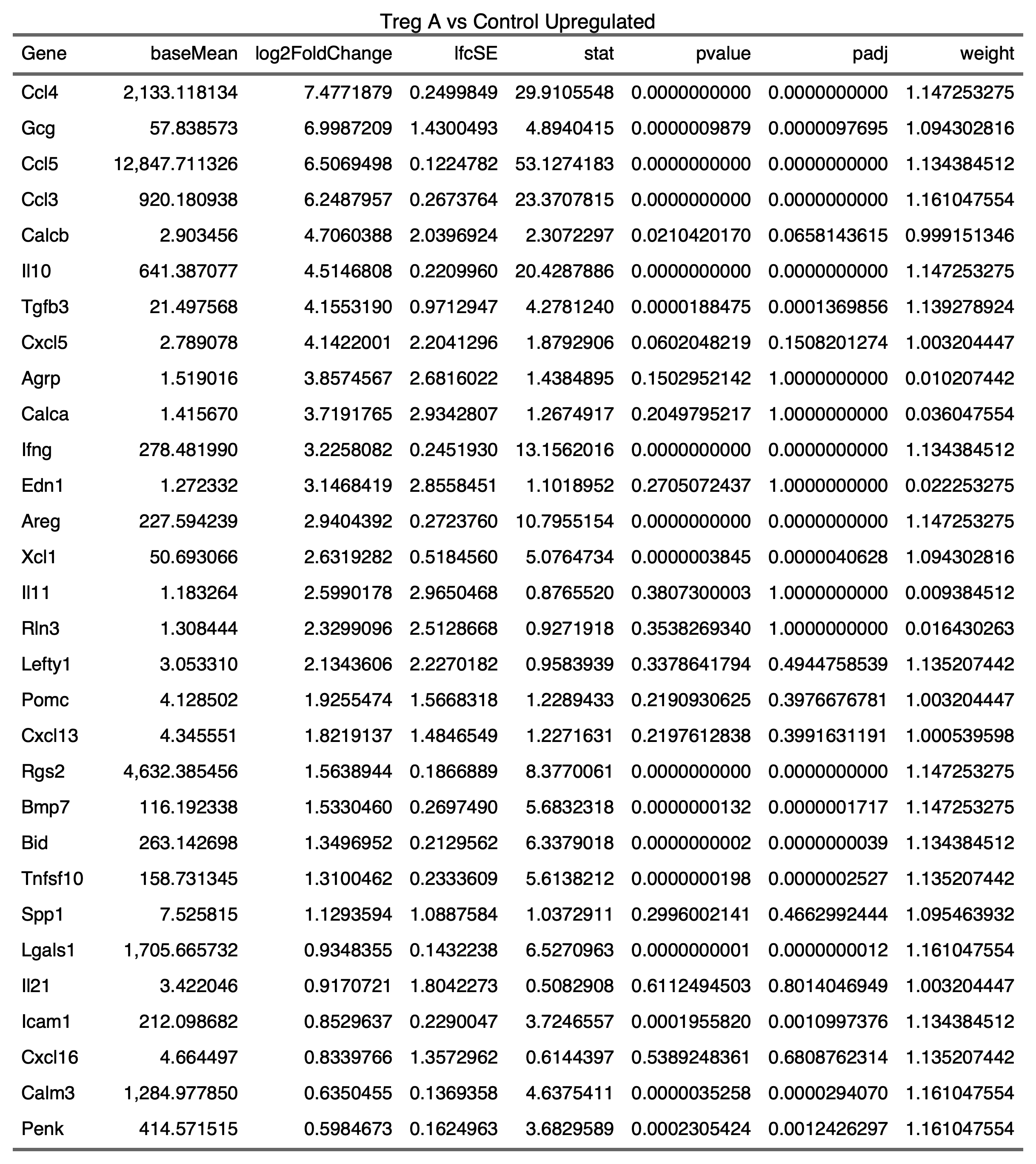


**Supp. Table 4**. The table with computed upregulated genes.
